# Supplementary material for: Effects of Three Interventions Combining Impact or Walking at Intense Pace Training, with or without Calcium and Vitamin Supplements, to Manage Postmenopausal Women with Osteopenia and Osteoporosis
Source: Int J Environ Res Public Health. 2022 Sep 7;19(18):11215. doi: 10.3390/ijerph191811215 (PMC9517092; doi:10.3390/ijerph191811215)
Supplement: Supplementary file 1 [file ijerph-19-11215-s001.zip › ijerph-1886792-supplementary.pdf]

DIAGNOSIS

	Group 1	Group 2	Group 3
DX femoral	6 normal<br>3 osteopenia<br>0 osteoporosis	11 Normal<br>5 Osteopenia<br>0 Osteoporosis	5 Normal<br>8 Osteopenia<br>0 Osteoporosis
DX lumbar spine	0 Normal<br>9 Osteopenia<br>0 Osteoporosis	2 Normal<br>10 Osteopenia<br>4 Osteoporosis	1 Normal<br>8 Osteopenia<br>5 Osteoporosis

0 Normal, 1 Osteopenia, 2 Osteoporosis

GROUP	DX femoral	DX spine
1	0	1
1	1	1
1	0	1
1	2	1
1	2	1
1	0	1
1	0	1
1	0	1
1	0	1
1	6 normal<br>3 osteopenia<br>0 osteoporosis	0 Normal<br>9 Osteopenia<br>0 Osteoporosis
2	0	1
2	0	1
2	0	2
2	0	1
2	0	2
2	0	2
2	1	0
2	1	0
2	1	1
2	0	1
2	0	1
2	0	1
2	0	1
2	0	1
2	1	2
2	1	1
2	11 Normal<br>5 Osteopenia<br>0 Osteoporosis	2 Normal<br>10 Osteopenia<br>4 Osteoporosis
3	0	2
3	1	2
3	1	2

3	0	1
3	1	2
3	1	1
3	1	1
3	1	1
3	0	1
3	0	2
3	0	1
3	1	0
3	1	1
3	1	1
3	5 Normal<br>8 Osteopenia<br>0 Osteoporosis	1 Normal<br>8 Osteopenia<br>5 Osteoporosis
